# A Protein-Linger Strategy Keeps the Plant On-Hold After Rehydration of Drought-Stressed *Beta vulgaris*

**DOI:** 10.3389/fpls.2019.00381

**Published:** 2019-03-29

**Authors:** Sebastian Schneider, Reinhard Turetschek, Rita Wedeking, Monika A. Wimmer, Stefanie Wienkoop

**Affiliations:** ^1^Division of Molecular Systems Biology, Department of Ecogenomics and Systems Biology, University of Vienna, Vienna, Austria; ^2^Institute of Crop Science and Resource Conservation – Plant Nutrition, University of Bonn, Bonn, Germany; ^3^Environmental Safety/Ecotoxicology, Bayer AG, Crop Science Division, Monheim am Rhein, Germany; ^4^Institute of Crop Science – Quality of Plant Products, University of Hohenheim, Stuttgart, Germany

**Keywords:** drought stress and recovery, memory effect, *Beta vulgaris*, protein targets, plant proteomics, molecular phenotyping, transcript to protein

## Abstract

Most crop plants are exposed to intermittent drought periods. To cope with these continuous changes, plants need strategies to prevent themselves from exhaustive adjustment maneuvers. Drought stress recovery has been shown to be an active process, possibly involved in a drought memory effect allowing plants to better cope with recurrent aridity. An integrated understanding of the molecular processes of enhanced drought tolerance is required to tailor key networks for improved crop protection. During summer, prolonged periods of drought are the major reason for economic yield losses of sugar beet (*Beta vulgaris*) in Europe. A drought stress and recovery time course experiment was carried out under controlled environmental conditions. In order to find regulatory key mechanisms enabling plants to rapidly react to periodic stress events, beets were either subjected to 11 days of progressive drought, or were drought stressed for 9 days followed by gradual rewatering for 14 days. Based on physiological measurements of leaf water relations and changes in different stress indicators, plants experienced a switch from moderate to severe water stress between day 9 and 11 of drought. The leaf proteome was analyzed, revealing induced protein pre-adjustment (prior to severe stress) and putative stress endurance processes. Three key protein targets, regulatory relevant during drought stress and with lingering levels of abundance upon rewatering were further exploited through their transcript performance. These three targets consist of a jasmonate induced, a salt-stress enhanced and a phosphatidylethanolamine-binding protein. The data demonstrate delayed protein responses to stress compared to their transcripts and indicate that the lingering mechanism is post-transcriptionally regulated. A set of lingering proteins is discussed with respect to a possible involvement in drought stress acclimation and memory effects.

## Introduction

Drought stress is one of the main constraints to crop production worldwide. Different from arid climates, central Europe faces increasing occurrence of intermittent drought periods, not only in late summer, but also during the early development of crops. Thus rapid recovery after drought spells is one of the traits which may improve crop yield under conditions of repeated drought stress (Chaves et al., 2009).

Recovery of a plant includes the re-establishment of osmotic homeostasis, the repair of tissues damaged by oxidative stress, and the re-adjustment of the plant’s metabolism. During this process, physiological parameters and independent metabolic pathways seem to require different times for a full recovery, (Lehmann et al., 2012; Wedeking et al., 2017), suggesting pathway specific regulatory processes (Lyon et al., 2016). Several studies have shown that plants can also respond differently to repeated periods of stress and that the first stress period might trigger maintained metabolic and epigenetic rearrangements commonly known as “stress memory” (stress imprint) (Bruce et al., 2007; Ding et al., 2012). Such imprints include the accumulation of protective metabolites (Bhargava and Kshitija, 2013), signaling proteins or transcription factors (Conrath et al., 2006; Ramírez et al., 2015), the phosphorylation of key regulatory proteins such as MAPKs, chromatin marks or epigenetic modifications (Ding et al., 2012; Kinoshita and Seki, 2014), all of which may render the plant more “prepared” and thus more resistant if the stress recurs. Even though this acclimation may be beneficial under repeated stress, it certainly comes at the cost of reduced growth since metabolic energy has to be used for maintained metabolic adjustments. In other cases plant performance was also reduced by maintained lower rate of photosynthesis after recurring stress (Bruce et al., 2007). Whether an initial stress is thus beneficial or rather counterproductive with respect to crop yield is likely dependent on the severity and the number of times the stress recurs. Crisp et al. (2016) argue that most plants return to the pre-stress metabolic and physiological state (“resetting”) during recovery. However, other studies indicate distinct dynamics and independent regulation of recovery after drought stress (Lehmann et al., 2012; Lyon et al., 2016; Wedeking et al., 2017, 2018).

While several studies and reviews highlight the importance of maintained transcriptional alterations during recovery, relatively little is known about the imprint of abiotic stress on other omics levels such as proteins and metabolites (Fleta-Soriano and Munné-Bosch, 2016), and whether such imprints may be related to later responses of recurring stress.

In previous work using an almost identical experimental setup as in the present study, we observed that sugar beet did not fully recover physiologically within 10 days of re-watering after 9 d of drought stress (Wedeking et al., 2017). In a similar study, sugar beet metabolites mostly returned to control levels within 8 d of re-watering, but especially the normalization of amino acids was only transient and a second increase of amino acids occurred several days (7-8) into the recovery period, indicating a possible stress imprint that might be beneficial in upcoming stress events (Wedeking et al., 2018). In the current study, we used a proteomic approach with the aim to identify proteins involved in the recovery process and possibly in stress imprints. For selected target proteins, we compared the time course of protein and transcript levels during the recovery period. We wanted to test the hypothesis that some proteins might not directly follow the course of transcript levels and that these proteins could be interesting targets for stress memory effects.

## Materials and Methods

### Plant Culture and Sampling for Physiological Parameters and Selected Metabolites

**FIGURE 1:**
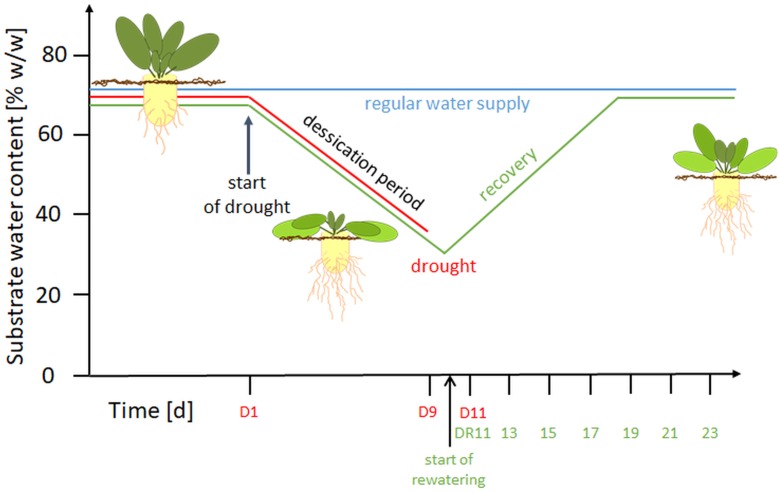
Overview of the experimental setup. D, drought; DR, drought recovery. Water withhold was from D1 until D9 or extended to D11. For the recovery experiment, rewatering started at day 10 until day 23.

Seedlings of *Beta vulgaris* cultivar Pauletta (KWS Saat AG, Einbeck, Germany) were grown under controlled conditions as described previously in Wedeking et al. (2017). Briefly, plants were cultivated at 24°C day/18°C night temperature, 60/75 % relative humidity and 16 h light (> 250 μmol m^-2^ s^1^: SON-T Agro, 400W, Philips, Germany) in a substrate mix (3:2:1 pea loam, perlit; Gepac, Type VM). Water and nutrients (1.4‰ Hakaphos blue, Compo Expert, Münster, Germany) were provided three times per day for 3 min, using a time controlled, automated irrigation system. Integrated plant protection was used as required. The experiment was arranged in a randomized block design with four biological replicates for each harvest day and treatment. A graphical overview of the experimental setup is provided in Figure 1.

Treatments began at BBCH 16-17 (Enz and Dachler, 1997) e.g., when 6 to 7 leaves were visible. For control plants (C), a soil water content (SWC) of 65 ± 1% (w/w; based on substrate FW), was maintained throughout the experiment. This SWC was previously shown to correspond to substrate pF values of 1.8-2.3 and thus represents optimum water supply (Wedeking et al., 2017). To confirm that neither water logging nor water deficit occurred during the experimental period, a subset of 15 pots was weighed manually every second day. Drought stressed plants (D) were subjected to 11 d of progressive drought by water withholding, while the plants of the recovery treatment (DR) were drought stressed for 9 or 11 with a subsequent gradual rewatering for 14 d (recovery period, day 10-23). In this setup, the SWC of drought stressed plants decreased to 45% (w/w) at around day 5 (corresponding to the permanent wilting point), and reached values of approximately 35% (w/w) on day 9, and 30% on day 11. During rewatering, the SWC increased linearly and reached control levels after 7-8 days (Supplementary Figure S1).

Plants were harvested every other day 4 h after the onset of the photoperiod to avoid uncontrolled circadian effects, e.g. of the water status and metabolite and protein concentrations. The youngest fully expanded leaf pair (YEL) was used for all analyses. One leaf was used for protein extraction, and the other one was used for the determination of osmotic potential (OP), relative water content (RWC) and electrolyte leakage (EL), as previously described in detail (Wedeking et al., 2017). Plant material for the protein and metabolite analysis was immediately frozen and ground in liquid nitrogen, lyophilized and stored (-80°C) until analysis. Leaf material for physiological measurements was frozen at -20°C (OP) or directly processed (RWC, EL).

For metabolite analysis, 20 mg of powdered material was sequentially extracted with 250 mm^3^ and 150 mm^3^ of 80% ethanol (v/v) in 10 mM HEPES KOH pH7, followed by 250 mm^3^ of 50% ethanol (v/v) in 10 mM HEPES KOH pH7 at 80°C for 20 min. The three supernatants were pooled and constantly kept on ice. Samples were analyzed using a microplate reader (Power Wave XS2, BioTek). Sucrose, glucose and fructose were determined using an enzyme-based assay according to Stitt et al. (1989) with modifications (for detailed description see Wedeking et al., 2017).

### Protein Extraction & Quantification

Total protein was extracted from 20 mg lyophilized ground leaf material according to Wang et al. (2006). Plant material was mixed with 10% trichloroacetic acid (TCA) in acetone and incubated for 10 min in a cold ultra-sonication water bath. With intermediate collecting steps by centrifugation at 4000 g for 5 min, the plant material was washed with 1.5 cm^3^ ice-cold 10% TCA in acetone, 1.5 cm^3^ ice-cold 10% TCA in water and 1.5 cm^3^ ice-cold 80% acetone. After the last centrifugation step, the supernatant was carefully discarded and the pellet was air dried. Proteins were extracted by vortexing washed plant material for 1 min at room temperature in 0.8 cm^3^ extraction buffer (50 mM TrisHCl pH 7.5, 5 mM EDTA, 0.7 M Sucrose, 1% PVPP (w/v), 1 mM PMSF, 5 mM DTT, ddH_2_O) and 0.8 cm^3^ phenol. For phase separation, the mixture was centrifuged at 10 000 g for 5 min. The protein containing phenol phase was transferred to a new tube. Phase separation was repeated and proteins were precipitated over night with 8 cm^3^ ice-cold 0.5% β-mercaptoethanol in acetone. Precipitated proteins were centrifuged for 10 min (4 000 g, 4°C). The supernatant was discarded and the protein pellet was washed two times with ice cold 100 mM ammonium acetate in methanol and two times with ice cold 80% acetone with intermediate centrifugation (20 000 g, 4°C, 5min).

### Digestion and Nano ESI LC-MS/MS Analysis

Proteins were digested and analyzed as previously described in Turetschek et al. (2017). The protein pellet was dissolved in urea buffer (8 M urea, 50 mM HEPES, pH 7.8) and quantified with Bradford assay. For each sample, 100 μg protein was digested with Lys-C (1:100 v/v, 5 h, 30°C, Roche, Mannheim, Germany) and trypsin (1:10, v/v, over- night, 37°C, Applied Biosystems, Darmstadt, Germany). The sample was acidified with 200 mm^3^ 8% formic acid (FA) and loaded on stage tips (Pierce^TM^ C18 Tips, 100 mm^3^). Peptides were washed 4 times with 200 mm^3^ 0.1% FA, eluted with 0.1% FA in methanol, split in two aliquots and stored at -80° in a protein LoBind tube until measurement.

Peptides of 4 biological replicates were dissolved in 100 mm^3^ 2% ACN, 0.1% FA. In random order 1 μg was applied on a C18 column (15 cm × 50 μm column, PepMap^®^RSLC, Thermo Scientific, 2 μm particle size) for separation during a 110 min gradient at a flow rate of 300 μm^3^ min^-1^. Measurement was done on an LTQ-Orbitrap Elite (Thermo Fisher Scientific, Bremen, Germany) with following settings: Full scan range 350–1800 m/z, max. 20 MS2 scans (activation type CID), repeat count 1, repeat duration 30 s, exclusion list size 500, exclusion duration 60 s, charge state screening enabled with rejection of unassigned and +1 charge states, minimum signal threshold 500.

### Protein Identification and Label Free Quantification

Proteins were identified and quantified as described in Turetschek et al. (2017) using a Uniprot FASTA download for *Beta vulgaris spp. vulgaris* with (29098 sequences feburary 2017) and the software MaxQuant v1.5_2 with the following parameters: first search peptide tolerance 20 ppm, main search tolerance 4.5 ppm, ITMS MS/MS match tolerance 0.6 Da. Maximum 3 of the following variable modifications was allowed per peptide: oxidation of methionine and acetylation of the N-term. Maximum two missed cleavages was tolerated. Best retention time alignment function was determined in a 20 min window. Identifications were matched between runs in a 0.7 min window. A FDR cut-off at 0.01 (at Peptide Spectrum Match and protein level) was set with a reversed decoy database. A minimum of 7 amino acids was required for identification of peptides and at least two peptides were required for protein identification. For label free quantification (LFQ) at least one MS2 scan was present with a minimum ratio of 2. The mass spectrometry proteomics data was deposited to the ProteomeXchange Consortium repository 3 with the dataset identifier PXD012033.

### Protein Target Selection

Three target proteins were selected for transcript analysis as they were statistically significantly more abundant (*p* < 0.05, ANOVA, fold change > 2 compared to control) during severe drought and along several days of recovery. Targets were identified and quantified solely through proteotypic peptides. Two housekeeping genes were also selected from the proteomics data as follows: proteins were checked for robustness (found in all samples) and stability (no statistically significant difference in abundance across all samples). Gene-specific primers for proteins were designed according to their genome sequences available in the *Beta vulgaris* uniprot database. All primers, including those for housekeeping’s and target genes, are listed in Supplementary Table S1.

### RNA Extraction

Total RNA was isolated from *B. vulgaris* leaf tissue with Trizol (Invitrogen^TM^ TRIzol^TM^ Reagent) according to manufacturer’s instructions. The RNA quality was assessed by 1% formaldehyde agarose gel electrophoresis and quantified spectrophotometrically.

### Reverse Transcriptase and Quantitative Real-Time qPCR

First-strand cDNA was synthesized from 2 μg of total RNA using Superscript First-Strand Synthesis System (Invitrogen, New York, United States).

Quantitative PCR was carried out using 3 biological and 3 technical replicates in a Mastercycler RealPlex (Eppendorf, Hamburg, Germany) using the iTaq Universal SYBR Green Supermix reagents (Bio-Rad). The program consisted of an initial denaturation and Taq polymerase activation step of 10 minutes at 95°C, 40 cycles of 15 s at 95°C, 1 minute annealing and elongation at 60°C, followed by a melting curve from 59°C to 95°C.

Quantitative analysis of target gene transcription was carried out using the 2exp(-ΔΔCT) method (Livak and Schmittgen, 2001).

### Statistical Analysis

For physiological data, statistical analysis was performed using SPSS 22.0 (SPSS Inc., IBM, United States). Significant differences between the treatments were analyzed using a one-factorial ANOVA according to Kruskal-Wallis (α = 0.05) with the stepwise stepdown procedure.

Statistical computation for proteins was done in R v3.4.3. Only proteins present in 3 out of 4 replicates in at least one treatment were considered for statistical analysis. If 3 out of 4 replicates in a treatment were present, missing values were estimated via k- nearest neighbor algorithm otherwise half the minimum value of the respective protein was inserted. Significant differences between groups were determined with Kruskal-Wallis test. All *p*-values were corrected according to Benjamini-Hochberg method. Significant proteins required a minimum fold change of ≥ 2 for further interpretation. Proteins were functionally classified with the software Mercator 4 platform^1^.

## Results

### Time Course of Physiological and Metabolic Responses

On day 9 of the drought stress (D9), osmotic potential and relative water content were significantly lower, and electrolyte leakage was significantly higher than the controls. These changes strongly intensified between D9 and D11 of drought (Supplementary Figure S1; see also Wedeking et al., 2017). All three parameters started to recover immediately upon rewatering (DR11), even though they all returned to control levels only on day DR17 (RWC, OP) or DR19 (EL). Hexoses (sum of glucose and fructose) were significantly reduced compared to controls, while sucrose was significantly increased during severe stress (D11). Different from physiological parameters, both metabolites did not start to return to the control level upon rewatering, but remained elevated during recovery (DR11 to DR15) and reached their highest values on day DR15.

### Unbiased Protein Dynamics Analysis

**FIGURE 2:**
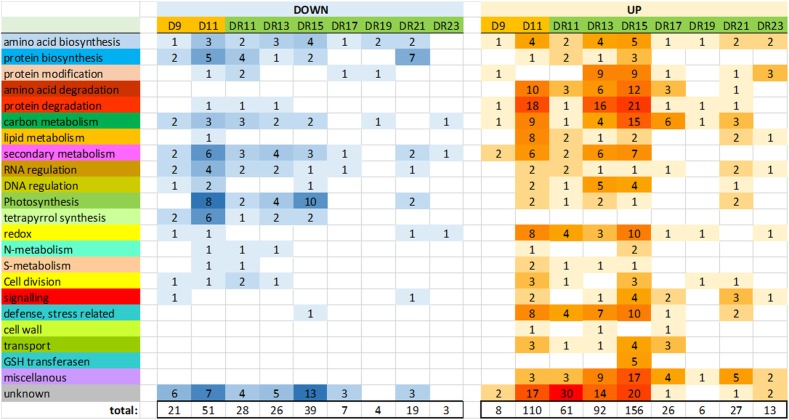
Overview of the number of proteins of major functional (MapMan) categories, which were significantly (*p* < 0.05) reduced (down) or increased (up) compared to controls.

Between drought day 9 (D9) and day 11 (D11), the number of significantly (*p* < 0.05) changed protein levels increased from 8 to 108 proteins (accumulated) and from 20 to 50 proteins (reduced), indicative for a transition from moderate to severe drought stress (Supplementary Figure S2 and Supplementary Table S2). With regard to gene ontologies, the groups “amino acid and protein degradation”, “carbon metabolism”, “lipid metabolism”, “defense/stress” and “redox” were specifically enhanced, while “photosynthesis”, “tetrapyrrolbiosynthesis” and “secondary metabolism” were reduced on day D11. The function of a relatively large number of enhanced proteins could not be identified (“unknown”) (Figure 2).

Interestingly, the number of significantly changed protein levels was also increased from day D9 of drought (29) to day 11 of the drought recovery, i.e., one day after the end of drought exposure (DR11: 89). Among the 144 different proteins that significantly accumulated at day 11 under drought (D11) or recovery (DR11), 27 were overlapping (Supplementary Figure S2).

Surprisingly, the number of significantly changed proteins did not continuously decrease throughout the recovery period. Rather, it strongly increased between day DR11 (89) and DR15 (195) of recovery, before it started to decrease (Figure 2). The GO groups which were most strongly affected on day DR15 were quite similar to those affected under severe drought on day D11, and they belonged to the categories “amino acid and protein degradation”, “carbon metabolism”, “secondary metabolism”, “redox”, and “defense/stress” (all increased), and “photosynthesis” (reduced) (Figure 2). Comparison of significantly changed proteins on days D11, DR11 and DR15 also indicates that the proteome on day DR15 is more similar to that on day D11 (severe stress) than that on day DR11 (beginning of recovery). However, there were also some distinct differences between D11 and DR15, e.g., we did not observe an increase in the functional group “lipid metabolism” on DR15, indicating that membrane damage did not occur to a similar extent as at D11. A very specific response on day DR15 was the increase in 5 different GSH transferases, which was not observed on any of the other days. These GSH transfereases might be part of a glutathione and ABA mediated signal transduction pathway during drought stress acclimation (Chen et al., 2012). From all significantly changed proteins on days D11, DR11 and DR15, there was a 26% overlap between D11 and DR15 for increased, and 23% overlap for reduced proteins (Supplementary Figure S2).

In the course of recovery (from DR11 until DR23), most proteins returned to control levels after 9 days of recovery (DR19), when only 10 proteins were detected which were significantly different from the controls. However, this was followed by a remarkable increase in significantly changed proteins (19 reduced and 27 increased) on day DR21. Reduced proteins mainly belonged to the functional group “amino acid and protein biosynthesis”, while increased proteins belonged to the groups “signaling”, “carbon metabolism”, “RNA regulation”, “photosynthesis” and “defense/stress” and “miscellanous”. The increased level of significantly changed proteins was only transient and was no longer observed on day DR23.

### Multivariate Statistics

An unbiased approach was initially used to detect proteins that might be key-players involved in drought stress recovery regulation. Using multivariate statistics, it was also possible to forecast proteins involved in dynamic stress adaptations that were distinct from general leaf senescence process.

**FIGURE 3:**
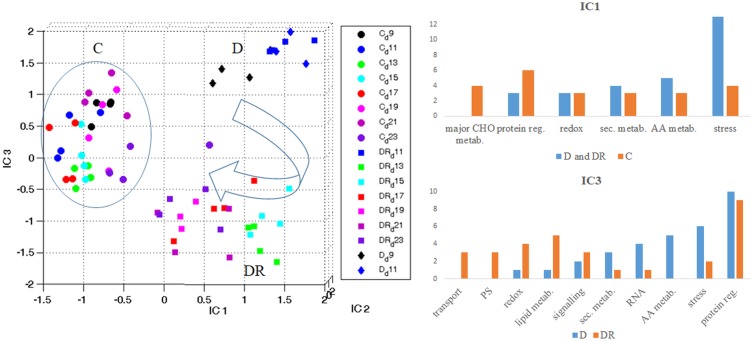
Overview of the protein stress and recovery trajectory. Independent component analysis (ICA) of all quantifiable leaf proteins of *B. vulgaris* control (C) plants as well as plants exposed to drought (D) and recovery (DR) at different days during treatment (9-23). IC1 separates proteins according to stress severeness (C versus D and DR). Functional categories of protein groups of highest loadings (IC1) are visualized in bar-plots (see also Supplementary Table S3a). IC3 separates proteins mostly involved in stress from those of recovery (D versus DR). Functional categories of protein groups with highest loadings (IC3) are visualized in bar-plots (see also Supplementary Table S3b).

When visualizing all quantifiable proteins by Independent Component Analysis (ICA) (Figure 3), IC1 clearly separates controls from drought stressed (and recovered) plants. IC1 likely represents stress severity, since severe drought (D11) and the first days of recovery (DR11-DR15) are furthest from controls, while later days of recovery (DR17-DR23) and moderate stress (D9) are closer. Interestingly, recovered plants on day DR23 are still clearly distinct from controls with respect to their proteome. IC3 separates drought stress from recovery. This plot indicates that drought stress at day 9 (D9) and day 11 (D11) are distinct from each other, while proteins of day 11 of recovery (DR11) appear indifferent from drought stress at day 11 (Figure 3). Altogether drought recovery is following a trajectory toward controls where proteins at day 11, 13 and 15 of recovery (DR11, DR13, DR15) remain at the far right side of IC1 together with day 11 of drought. Functional categorization (categories containing > 3 proteins; 100 highest loadings in the range of > 0.0045 and < -0.0045) of those proteins with increased levels upon stress (IC1), indicates a dominance of the stress responsive protein group, followed by the groups of amino acid metabolism, secondary metabolism, redox regulation and protein regulation (Figure 3 and Supplementary Table S3a). In contrast, proteins with decreased levels under stress belonged mainly to the categories protein regulation, followed by major carbon metabolism and stress responsive proteins (Figure 3 and Supplementary Table S3). The dominant category of the 100 proteins with highest loadings, separating drought from recovery (IC3), was the group of protein regulation in both depleted and accumulated proteins (Figure 3 and Supplementary Table S3b). Furthermore, among those proteins accumulated during drought, the second strongest category was stress regulation, followed by amino acid metabolism, RNA regulation, secondary metabolism, signaling, lipid metabolism and redox regulation. Apart from protein regulation, proteins specifically accumulated during recovery belonged to the category lipid metabolism, followed by redox regulation, photosystem, and transport and signaling protein groups.

### Targeted Protein and RNA Analysis

Multivariate statistics separated between controls, stress and recovery (Figure 3). In order to study those proteins involved in stress as well as recovery, all significantly changed proteins (compared to control) were selected in a first step. From this group, proteins of day 11 overlapping between drought and recovery (25) and those overlapping between day 11 and day 9 of drought (2), were filtered (Supplementary Figure S2 and Table 1).

**Table 1:** List of filtered target proteins significantly accumulating during recovery and possibly involved in drought stress imprint.

	Descriptions	Mapman	D11/C11	DR11/C11	DR13/C13	DR15/C15	DR17/C17	DR19/C19	DR21/C21	DR23/C23
A0A0J8D7Q4	Lea14 homolog	development	19^∗∗∗^	18^∗∗∗^	20^∗∗∗^	14^∗∗∗^	6	8	5	2
A0A0J8B6M2	Uncharacterized protein	not assigned	5^∗∗∗^	4^∗∗∗^	3^∗∗^	3^∗∗^	2	1	2	1
A0A0J8F7N2	similar to salt-induced protein	Stress	9^∗∗∗^	9^∗∗∗^	6^∗^	2	2	1	1	1
A0A0J8CII1	Uncharacterized protein	not assigned	279^∗∗∗^	244^∗∗∗^	3	128	128	6	92	2
A0A0J8CBL0; A0A0J8C792	Uncharacterized protein	not assigned	7^∗∗∗^	^∗∗∗^	1	1	2	1	1	1
A0A0J8CRM6	putative jasmonate-induced protein	Stress	4^∗∗^	6^∗∗∗^	2	2	2	1	1	1
A0A0J8CDM0	Uncharacterized protein	not assigned	4^∗∗^	4^∗∗∗^	1	1	1	1	1	1
A0A0J8ER97	vacuolar ATP synthase subunit D	Transport	2^∗^	^∗∗∗^	1	1	1	1	1	1
A0A0J8CUI5	Lea protein	development	2^∗^	2^∗∗∗^	2^∗∗∗^	1	1	1	1	1
A0A0J8BLJ1	Octicosapeptide domain protein	not assigned	3^∗∗^	3^∗∗^	3^∗∗∗^	4^∗∗∗^	2	1	1	1
A0A0J8BAX7	Formate dehydrogenase	C1-metabolism	2^∗∗∗^	2^∗^	2^∗∗∗^	2^∗∗∗^	1	1	1	1
A0A0J8EVD3	Uncharacterized protein	not assigned	3934^∗∗∗^	2566^∗∗^	1357	2954^∗∗∗^	865	219	6	2
A0A0J8BFR5	Uncharacterized protein	Metal handling	4^∗∗∗^	3^∗∗^	4^∗∗^	5^∗∗^	2	1	1	1
A0A0J8CLR7	Thiolase family protein	AA metabolism	11^∗∗∗^	8^∗^	57^∗∗^	80^∗∗^	4	1	1	2
A0A0J8FDN2; A0A0J8FDM4	dioxygenase LigB subunit	not assigned	348^∗∗∗^	249^∗∗^	125^∗^	18^∗∗∗^	3	38	3	11
A0A0J8CMM2	Adenine nucleotide alpha hydrolase	Stress.abiotic	3^∗∗∗^	2^∗∗^	2^∗^	4^∗∗∗^	2	1	1	1
A0A0J8CG86	aspartyl proteaseprotein	RNA	7^∗∗∗^	4^∗^	3^∗^	2	0	1	1	1
A0A0J8BGX7	3-methylcrotonyl-CoA carboxylase	AA metabolism	66^∗^	57^∗^	65	134^∗∗∗^	92^∗∗^	0	8^∗^	11
A0A0J8ELN5	ornithine delta-aminotransferase	AA metabolism	289^∗∗∗^	182^∗∗^	14	20^∗∗^	3	1	4	1
A0A0J8CFP3	glutathione transferase	miscellaneous	3^∗∗^	3^∗^	1	3^∗∗^	1	0	1	1
A0A0J8BGE0	aspartate aminotransferase	AA metabolism	419^∗∗∗^	243^∗∗^	68	79	56	42	57	1
A0A0J8C766; A0A0J8F1S1	Uncharacterized protein	not assigned	12^∗∗∗^	7^∗^	1	3	2	1	1	1
A0A0J8CZJ5; A0A0J8CUA1	Uncharacterized protein	not assigned	13^∗∗^	11^∗^	1	2	1	1	1	1
A0A0J8B2R5	Uncharacterized protein	not assigned	3^∗∗^	3^∗^	2	2	2	1	1	2
A0A0J8CT86	Uncharacterized protein	not assigned	14^∗^	15^∗^	1	1	1	1	1	42
A0A0J8BRE3	3-Phosphoglycerate dehydrogenase	AA metabolism	5^∗∗∗^	5^∗∗∗^	2^∗∗∗^	2	1	1	1	1
A0A0J8F906	PEBP	not assigned	3^∗∗∗^	3^∗∗∗^	4^∗∗∗^	3	2^∗∗^	1	1	1


*n = 4; Asterisks ^∗^p < 0.05, ^∗∗^*p* < 0.001; ^∗∗∗^*p* < 0.0001.*

**FIGURE 4:**
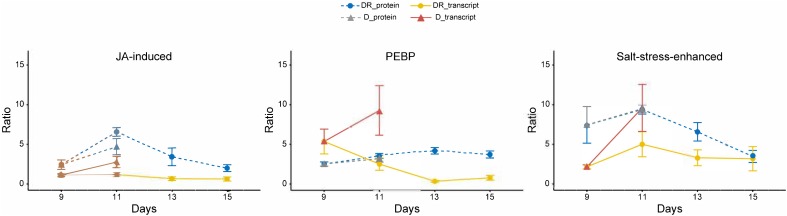
Time course of transcript and protein targets. Ratios (controls vs. treatment); statistics (Kruskal-Wallis) for protein targets are given in Table 1; *n* = 4; error bars, standard error. Transcripts ratios are significant (*p* < 0.05; Kruskal-Wallis) at D11 of all targets and at D9 of “salt stress enhanced” protein.

Three proteins that showed highest statistically significant accumulation during severe drought (D11) (*p* < 0.005) as well as recovery (DR11) (*p* < 0.00005) were selected from the datamatrix (Supplementary Table S2). All three proteins were already enhanced at moderate drought (D9), even though this increase was only significant for phosphatidylethanolamine-binding protein (PEBP, A0A0J8F906). In addition, this protein was significantly (> 2-fold) enhanced for the longest period of recovery, until DR 17 (7 days after the onset of rewatering) (Figure 4). The other two proteins (A0A0J8CRM6, a putative jasmonate-induced and A0A0J8F7N2, a protein similar to a salt stress-induced protein) were more drastically enhanced in abundance (up to 10-fold) compared to PEBP, especially at day 11 (D11) of severe drought and at DR11. Both remained elevated until DR13 (A0A0J8F7N2) or DR11 (A0A0J8CRM6). All three proteins are commonly found in high loadings lists of IC1 (Supplementary Table S3) separating stress from control.

Following the time course, all three targets showed a significant induction of protein and transcript abundance on day D11 of drought stress. This was also observed on day DR11 of recovery for A0A0J8F7N2, whereas A0A0J8CRM6 and PEBP showed an opposite reaction for proteins and transcripts on this day, where protein levels were still significanly higher compared to day D9, while transcripts had started to decline. The difference in mRNA levels between D11 and DR11 (always higher on D11) were not reflected at corresponding levels for proteins, which were not significantly different between days D11 and DR11, indicating that transcript levels responded more quickly to the rewatering signal compared to the proteins. A negative correlation (*r*^2^ = 0.7) between transcript (returning to control) and protein (remaining enhanced) development was especially pronounced for PEBP, which was also the only target whose transcript levels were accumulated to a higher degree (10-fold) compared to the protein levels (4-fold) on D11 of drought. The opposing trend for transcripts and proteins during recovery indicates a delayed translation for at least 4 days into the rewatering period for PEBP, and to a slightly lower extent also for A0A0J8CRM6.

## Discussion

### Recovery Upon the Onset of Severe Drought Involves Deceleration of Induced Stress Response Processes

Stress recovery and stress imprint involves reprogramming on multiple levels comprising physiological, transcriptional, proteomic and metabolic processes as well as epigenetics (Kinoshita and Seki, 2014). These effects may be triggered at different stages during moderate stress and may persist for different periods of time after priming. This prepares the plants to promptly respond to reiterated drought (Walter et al., 2011; Ramírez et al., 2015). Since the occurrence and intensity of a possible imprint is likely to depend on the initial stress level at the onset of recovery, it is important to choose a well-defined experimental setup where the physiological stress level is well established. Using an almost identical setup, we have previously shown that sugar beets respond to a transient drought stress first by stomatal closure and cessation of transpiration, followed by adaptive metabolic adjustments such as increased sucrose, malate or proline levels, which may help to cope with reduced water availability by redirecting carbon flow into pathways relevant for protective responses. Between day 9 and 11 of drought stress, plants were in a transition from moderate to severe drought stress (Wedeking et al., 2017). Therefore, the current study focused on the determination of leaf proteins of *B. vulgaris*, involved in recovery and to identify putative candidates for future studies on memory-effects by a comprehensive analysis of the proteome characteristics during a long period of recovery (13 days) after 9 d of drought.

Stress recovery is an important molecular process that needs further attention to comprehensively understand priming effects leading to improved stress tolerance. In recent studies on *Medicago truncatula* and *B. vulgaris*, drought stress recovery was shown to be an active and highly dynamic process at both protein and metabolite level (Lyon et al., 2016; Wedeking et al., 2017).

Multivariate statistics detangled specific functional protein categories that differentiate drought and drought recovery from control plants. Not surprisingly, the major category involved proteins of the stress response, such as heat shock and LEA proteins as well as several salt- and jasmonate-induced proteins. One of the most unexpected results was the relatively large overlap of significantly enhanced proteins under severe stress (D11) and after one day of rewatering (DR11), and the intensification of the number of responsive proteins during the first 5 days of recovery (until DR15). Many altered proteins on day DR15 overlapped with those enhanced on day D11 (severe stress), even though the intensities of change were overall less pronounced at DR15. This suggests an induction of several proteins already during the drought stress, which accumulated with a delay of up to 4 days compared to the severe drought stress. However, since these proteins responded with an overall lower intensity on day DR15, the proteome of DR11 was statistically more similar to D11 than to DR15 (Figure 3). While it was shown in a previous study, that sugar beet leaves regained almost full turgescence within 24 h after re-watering, indicating a very rapid onset of recovery of water relations (Wedeking et al., 2017), stress responses such as osmotic potential, relative water content and most primary metabolites returned to control levels more slowly but within several days of recovery (Supplementary Figure S2). There was no indication that plants experienced an increased stress level between day DR11 and DR15 in terms of physiological parameters. It therefore seems that the present results indicate a surprisingly long lagging/lingering of stress-induced proteins during the first days of recovery. Nevertheless, the number of responsive proteins was by far the highest at DR15 (also higher than at D11), indicating that additional processes were induced, which were relevant for the recovery. It is likely that the observed increases in protein levels between DR11 and DR15 reflect metabolic adjustments necessary for repair mechanisms, which then result in a normalization of most physiological and metabolic parameters at around day DR17 (see also Wedeking et al., 2017, 2018). Specifically, the group “lipid metabolism” was almost normalized at DR15, which is in line with a normalization of leakage and MDA content observed in a previous study (Wedeking et al., 2017). A strong activation of metabolism during this period of recovery is also indicated by an increase of proteins in the categories transport, RNA regulation, redox and signaling. A transient increase in hexoses between DR11 and DR15 (Supplementary Figure S2), might also indicate activation of some sugar-responsive metabolic processes, and correlate with elevated levels of, e.g., a glutamine synthetase isozyme (EC 6.3.1.2), which is known to be responsive to sucrose stimulus (Sahrawy et al., 2004). Finally, we observed an increase in GSH transferases only on day DR15. These GSH transferases might be a part of a glutathione and ABA mediated signal transduction pathway involved in drought stress acclimation (Chen et al., 2012). Taken together, we found that the alteration of several proteins was already triggered before the onset of recovery, and within the transition from moderate to severe stress. Recovery after the onset of severe drought involved deceleration mechanisms from stress induction. The duration of this process might set a frame for the time required for full recovery and possible implications for subsequent stress responses.

A transient peak in the amount of altered proteins occurred on day DR21 of recovery. It was not dominated by a specific class of proteins, but included several proteins related to protein synthesis, signaling, RNA regulation, carbon metabolism as well as stress-related proteins. The fact that an identical transient peak was observed in a previous independent study on metabolites (Wedeking et al., 2018), suggests that this was a reproducible and active response not related to an uncontrolled external stress factor (e.g., pathogens). The simultaneous transient increase in hexoses and sucrose (Supplementary Figure S1) might suggest that the observed changes were related to the onset of re-growth. Experiments are under way to investigate the possibility that this transient activation of the metabolism during a later stage of recovery could also be a relevant component of a memory event.

### Lingering Proteins Seem to Have Slow Turnover Rates and Are Not Rapidly Induced

For a targeted analysis, we focused on leaf proteins of *B. vulgaris* that were induced upon drought and remained significantly (*p* < 0.05) enhanced or even further increased over several days after rewatering, indicating an important role during drought recovery (Table 1). Notably, several proteins were also negatively affected (depleted) upon severe stress and recovery and may also be important stress regulators. Nevertheless, we selected targets that remained accumulated during drought recovery as their profiles were expected to be easier to track. Overall, 25 proteins significantly (*p* < 0.05) increased on day 11 of drought (D11) as well as 1 day after recovery (DR11). This observation is somewhat surprising, since previous studies have shown that sugar beet plants regain turgescence and clearly start to visually recover within 24 h of rewatering (Wedeking et al., 2017). One might therefore expect an improvement for all proteins at day 11 of recovery compared to the continued and even more severe drought at day 11. Hence, this is a first indication that gene regulation of these proteins was already induced earlier during drought (on or before day D9) as they were simultaneously found accumulating during day 11 of drought and recovery. In order to further understand the regulation of these proteins and to see whether gene induction occurred earlier, we selected three proteins significantly enhanced during drought and with different lingering performance after rewatering: 1) a phosphatidylethanolamine-binding protein (A0A0J8F906), which was already responsive at mild drought and remained significantly accumulated for the longest period during recovery, until DR17; 2) a protein similar to a salt induced protein (A0A0J8F7N2) which was most strongly enhanced during drought and remained significantly accumulated until DR13 and 3) a putative jasmonate-induced protein homolog (A0A0J8CRM6) that was lingering until DR11 with significantly increased levels. For those targets, transcript levels were investigated and profiles compared with protein abundances.

Previously, we discussed challenges of correlating transcript and protein data (Stare et al., 2017). When studying the responses to a potato virus in leaves, several mRNA and protein levels were found not well correlated, mostly concerning the strength of response. While in the previous study stronger effects were found in abundance changes for mRNA levels compared to the proteins (Stare et al., 2017), the target proteins in the present study were induced more strongly, for a longer period of time, but also delayed compared to their transcripts. All these data demonstrate the limitation of transcript data alone, especially when searching for lasting effects where the duration of a changed protein level seems of key importance. Thus, induction of transcripts and proteins during stress acclimation processes can vary in time and intensity, which favors the study of protein levels rather than transcripts in order to directly see the regulator abundance and ideally also its activity and posttranslational status if applicable.

Protein A0A0J8F7N2 is similar to a salt induced hydrophilic protein of the natural halophytes *Suaeda glauca* and *Atriplex nummularia* and thus presumably involved in salt resistance (Tabuchi et al., 2005; Jin et al., 2016). Our data suggest that it is significantly enhanced upon severe drought and not only salt and can therefore be considered a more general abiotic stress marker. While protein levels of this protein remained significantly elevated until day DR13, transcript levels were only significantly (*p* < 0.05) enhanced at D11, indicating a stronger accumulation of the protein, which might be explained by a lower degradation to synthesis rate compared to transcripts. Although, the mRNA levels were significantly increasing at severe drought (D11) compared to D9, this increase was not higher at protein level compared to D9, indicating a delayed protein translation with the onset of severe drought. Hence, this salt stress-induced protein was already drought induced earlier and its delayed accumulation is reflected by constant levels from D9 to D11. Further experiments are needed, however, in order to test whether endurance and accumulative properties might play a role in short drought period reiterations.

The protein sequence of A0A0J8CRM6 is similar to a jasmonate (JA) induced protein described for *Atriplex canescens*, an indeciduous shrub. Several homologs of JA induced proteins, which are enhanced during drought, can be found in the high loadings list of IC1 together with several late embryogenesis abundant proteins within the most prominent functional category stress (Supplementary Table S3). However, this protein was the only one of all JA induced isoforms that remained elevated for several days after rewatering. JA is involved in many regulatory processes such as abiotic and biotic stress responses (Sofo et al., 2016). As a phytohormone, it seems evident that minor changes in JA concentration must lead to a fast response of the transcript and subsequently the metabolism. Here, the transcript levels significantly but transiently increased at D11, implying that JA induced responses were triggered under severe stress. In contrast, protein levels were accumulated significantly and to a higher degree on day D11 and reached their peak only on day DR11, indicating that an initial signaling must have occurred during the onset of severe stress (probably at day 10) and thus transcript and protein were simultaneously induced before day 11 and regardless of the varying water status at day 11. It seems that overall, the phytohormone (JA) to protein signaling, for both induction as well as degradation, occurred more rapidly, compared to the other targets. This is further supported by the fact that this protein returned to control levels fastest and was not significantly accumulated from D13 onwards. Hence, this protein seems to be a quick responder and might therefore be rather involved in signaling of severe drought stress.

A totally different response pattern was observed for the third target protein A0A0J8F906, representing a phosphatidylethanolamine-binding protein (PEBP). The delayed accumulation and depletion (negative transcript to protein correlation) of this protein with respect to its mRNA indicates a slow protein turnover process described by delayed synthesis as well as degradation rates. PEBP is a serine protease inhibitor (Hengst et al., 2001) that is involved in signaling mediation via association with phosphorylated proteins in tomato (Pnueli et al., 2001). These findings support its involvement in post-translational regulation. It was also reported to control flowering and stem architecture (Banfield et al., 1980) and the timing of the switch from vegetative to reproductive development (Bäurle and Dean, 2006). We found it only slightly decreasing with age in the leaves of control plants (d9 – d23), but as to how this might be related to the timing of development remains unclear. Interestingly, PEBP is also associated with a drought stress responsive gene-network in rice (Smita et al., 2013). Compared to the other two target proteins, PEBP remained significantly (*p* < 0.05) accumulated until DR17, possibly indicating that it is a regulator specifically during recovery. Furthermore, PEBP might be a good candidate for further studies on stress memory effects leading to improved drought tolerance.

These three candidates have been chosen randomly from those that were functionally characterized in the target list (Table 1). However, other targets of this list, such as late embryogenesis abundant (Lea) proteins, are also well known drought responsive proteins (Magwanga et al., 2018). In addition, a gene of an Octicosapeptide/Phox/Bem1p (PB1) domain-containing protein has been described to be drought responsive and regulated by at least three plant hormones (Huang et al., 2008). The function of most of these target proteins have, however, thus far not been characterized. Hence, the findings of this study strongly indicate that these proteins are involved in drought stress regulation, especially during recovery.

Altogether, our data indicate that lingering proteins, such as PEBP, seem to have a rather slow turnover rate leading to decelerated accumulation and degradation compared to their transcripts. This would explain why some proteins involved in drought stress recovery (or even stress memory) may only be found by proteome analysis, because they are not well or even negatively correlated to their transcript levels.

## Conclusion

Taken together, we found evidence for a delayed protein response at the transition from moderate to severe drought stress, which seems to be preassigned at genome/transcript level already day(s) before. This delayed protein response reaches similar intensities at severe drought and initially after recovery and is, hence, independent on transcript levels and the actual water status of the plant. After rewatering, induced stress response processes are decelerated. This includes that several proteins remain in position or increase/decrease even further for a period of several days after recovery before their levels return to control levels. We propose that some of these lingering proteins might be involved in drought stress memory effects. The approach used in this study, applying in depth phenotyping along a drought stress transition state by integrating different techniques, is thus far unique. Hence, the suite of proteins, presented here, are interesting candidates for future experiments on reiterative drought stress scenarios and for studying the functions and regulatory processes involved in stress recovery and possible stress imprints.

## Data Availability

The mass spectrometry proteomics data have been deposited to the ProteomeXchange Consortium via the PRIDE (Vizcaíno et al., 2016) partner repository (https://www.ebi.ac.uk/pride/archive/) with the dataset identifier PXD012033.

## Author Contributions

MW, RW, and SW conceived the study. RT and SW performed proteomics and data analysis. SW was extracting RNA and chose targets and primer. SS conducted the qPCR. MW and RW performed plant experimental setup and physiological measurements and wrote the manuscript. SW was involved in planning and supervised the work and took the lead in writing the manuscript. All authors provided critical feedback and helped shape the research, read and approved the manuscript.

## Conflict of Interest Statement

The authors declare that the research was conducted in the absence of any commercial or financial relationships that could be construed as a potential conflict of interest.

## Footnotes

1 http://www.plabipd.de/portal/mercator-sequence-annotation

## Supplementary Material

FIGURE S1 Time course of different physiological parameters (RWC: relative water content; OP: osmotic potential; EL: electrolyte leakage) and metabolites (hexoses: sum of fructose and glucose) during stress and recovery. Values represent ratios of stressed to control plants of the same day. Asterisks indicate statistically significant differences (controls versus treatment), ^∗^*p* < 0.05 (Kruskal-Wallis), *n* = 4.

FIGURE S2 Venn diagrams of the numbers of statistically (*p* < 0.05; Kruskal-Wallis) significantly changed proteins overlapping between D9, D11 and DR11.

TABLE S1 Information about the selected RNA primers of the target proteins used for qPCR.

TABLE S2 *Beta vulgaris* leaf proteomics data. Identified proteins, mapped functional categories, ratio of mean LFQ intensities per paired comparison of treatments (*n* = 4) and ANOVA (*p*-val < 0.05 green; FC–ratios > 2 red; FC–ratios < 2 yellow ); “not valid” if too many missing values for statistics in one of the treatments (*n* < 4).

TABLE S3 List of highest loadings for IC1 (a) and IC3 (b) corresponding to ICA, Figure 3.

## References

[B1] Banfield M. J. Barker J. J. Perry A. C. F. Brady R. L. (1980). Function from structure? The crystal structure of human phosphatidylethanolamine-binding protein suggests a role in membrane signal transduction. *Structure* 6 1245–1254. 9782050 10.1016/s0969-2126(98)00125-7

[B2] Bäurle I. Dean C. (2006). The timing of developmental transitions in plants. *Cell* 125 655–664. 10.1016/j.cell.2006.05.005 16713560

[B3] Bhargava S. Kshitija S. (2013). Drought stress adaptation: metabolic adjustment and regulation of gene expression. *Plant Breed.* 132 21–32. 10.1111/pbr.12004

[B4] Bruce T. J. A. Matthes M. C. Napier J. A. Pickett J. A. (2007). Stressful “memories” of plants: evidence and possible mechanisms. *Plant Sci.* 173 603–608. 10.1016/j.plantsci.2007.09.002

[B5] Chaves M. M. Flexas J. Pinheiro C. (2009). Photosynthesis under drought and salt stress: regulation mechanisms from whole plant to cell. *Ann. Bot.* 103 551–560. 10.1093/aob/mcn125 18662937 PMC2707345

[B6] Chen J.-H. Jiang H.-W. Hsieh E.-J. Chen H.-Y. Chien C.-T. Hsieh H.-L. (2012). Drought and salt stress tolerance of an *Arabidopsis* glutathione S-transferase U17 knockout mutant are attributed to the combined effect of glutathione and abscisic acid. *Plant Physiol.* 158 340–351. 10.1104/pp.111.181875 22095046 PMC3252094

[B7] Conrath U. Beckers G. J. M. Flors V. García-Agustín P. Jakab G. Mauch F. (2006). Priming: getting ready for battle. *Mol. Pant Microbe Interact.* 19 1062–1071. 10.1094/MPMI-19-1062 17022170

[B8] Crisp P. A. Ganguly D. Eichten S. R. Borevitz J. O. Pogson B. J. (2016). Reconsidering plant memory: intersections between stress recovery, RNA turnover, and epigenetics. *Sci. Adv.* 2:e1501340. 10.1126/sciadv.1501340 26989783 PMC4788475

[B9] Ding Y. Fromm M. Avramova Z. (2012). Multiple exposures to drought “train” transcriptional responses in *Arabidopsis.* *Nat. Commun.* 3:740. 10.1038/ncomms1732 22415831

[B10] Enz M. Dachler C. (1997). *Compendium of Growth Stage Identification Keys for Mono-and Dicotyledonous Plants–Extended BBCH Scale* 2nd Edn Leverkusen: Bayer.

[B11] Fleta-Soriano E. Munné-Bosch S. (2016). Stress memory and the inevitable effects of drought: a physiological perspective. *Front. Plant Sci.* 7:143. 10.3389/fpls.2016.00143 26913046 PMC4753297

[B12] Hengst U. Albrecht H. Hess D. Monard D. (2001). The phosphatidylethanolamine-binding protein is the prototype of a novel family of serine protease inhibitors. *J. Biol. Chem.* 276 535–540. 10.1074/jbc.M002524200 11034991

[B13] Huang D. Wu W. Abrams S. R. Cutler A. J. (2008). The relationship of drought-related gene expression in *Arabidopsis thaliana* to hormonal and environmental factors. *J. Exp. Bot.* 59 2991–3007. 10.1093/jxb/ern155 18552355 PMC2504347

[B14] Jin H. Dong D. Yang Q. Zhu D. (2016). Salt-responsive transcriptome profiling of suaeda glauca via RNA sequencing. *PLoS One* 11:e0150504. 10.1371/journal.pone.0150504 26930632 PMC4773115

[B15] Kinoshita T. Seki M. (2014). Epigenetic memory for stress response and adaptation in plants. *Plant Cell Physiol.* 55 1859–1863. 10.1093/pcp/pcu125 25298421

[B16] Lehmann M. Laxa M. Sweetlove L. J. Fernie A. R. Obata T. (2012). Metabolic recovery of *Arabidopsis thaliana* roots following cessation of oxidative stress. *Metabolomics* 8 143–153. 10.1007/s11306-011-0296-1 22279429 PMC3258409

[B17] Livak K. J. Schmittgen T. D. (2001). Analysis of relative gene expression data using real-time quantitative PCR and the 2-ΔΔCT method. *Methods* 25 402–408. 10.1006/METH.2001.1262 11846609

[B18] Lyon D. Castillejo M. A. Mehmeti-Tershani V. Staudinger C. Kleemaier C. Wienkoop S. (2016). Drought and recovery: independently regulated processes highlighting the importance of protein turnover dynamics and translational regulation. *Mol. Cell. Proteomics* 15 1921–1937. 10.1074/mcp.M115.049205 27001437 PMC5083093

[B19] Magwanga R. O. Lu P. Kirungu J. N. Lu H. Wang X. Cai X. (2018). Characterization of the late embryogenesis abundant (LEA) proteins family and their role in drought stress tolerance in upland cotton. *BMC Genet.* 19:6. 10.1186/s12863-017-0596-1 29334890 PMC5769447

[B20] Pnueli L. Gutfinger T. Hareven D. Ben-naim O. Ron N. Adir N. (2001). Tomato SP-interacting proteins define a conserved signaling system that regulates shoot architecture and Flowering. *Plant Cell* 13 2687–2702. 10.1105/tpc.010293 11752381 PMC139482

[B21] Ramírez D. A. Rolando J. L. Yactayo W. Monneveux P. Mares V. Quiroz R. (2015). Improving potato drought tolerance through the induction of long-term water stress memory. *Plant Sci.* 238 26–32. 10.1016/J.PLANTSCI.2015.05.016 26259171

[B22] Sahrawy M. Ávila C. Chueca A. Cánovas F. M. López-Gorgé J. (2004). Increased sucrose level and altered nitrogen metabolism in *Arabidopsis thaliana* transgenic plants expressing antisense chloroplastic fructose-1,6-bisphosphatase. *J. Exp. Bot.* 55 2495–2503. 10.1093/jxb/erh257 15448173

[B23] Smita S. Katiyar A. Pandey D. M. Chinnusamy V. Archak S. Bansal K. C. (2013). Identification of conserved drought stress responsive gene-network across tissues and developmental stages in rice. *Bioinformation* 9 72–78. 10.6026/97320630009072 23390349 PMC3563401

[B24] Sofo A. Nahar K. Ahmad P. Rasool S. Gul A. Sheikh S. A. (2016). Jasmonates: multifunctional roles in stress tolerance. *Front. Plant Sci.* 7:813. 10.3389/fpls.2016.00813 27379115 PMC4908892

[B25] Stare T. Stare K. Weckwerth W. Wienkoop S. Gruden K. (2017). Comparison between proteome and transcriptome response in potato (*Solanum tuberosum* L.) leaves following potato virus Y (PVY) infection. *Proteomes* 5:14. 10.3390/proteomes5030014 28684682 PMC5620531

[B26] Stitt M. Lilley R. M. Gerhardt R. Heldt H. W. (1989). Metabolite levels in specific cells and subcellular compartments of plant leaves. *Methods Enzymol.* 174 518–552. 10.1016/0076-6879(89)74035-0

[B27] Tabuchi T. Kawaguchi Y. Azuma T. Nanmori T. Yasuda T. (2005). Similar regulation patterns of choline monooxygenase, phosphoethanolamine N-methyltransferase and S-adenosyl-L-methionine synthetase in leaves of the halophyte *Atriplex nummularia* L. *Plant Cell Physiol.* 46 505–513. 10.1093/pcp/pci050 15695433

[B28] Turetschek R. Desalegn G. Epple T. Kaul H.-P. Wienkoop S. (2017). Key metabolic traits of *Pisum sativum* maintain cell vitality during Didymella pinodes infection: cultivar resistance and the microsymbionts’ influence. *J. Proteomics* 169 189–201. 10.1016/j.jprot.2017.03.001 28268116

[B29] Vizcaíno J. A. Csordas A. Del-Toro N. Dianes J. A. Griss J. Lavidas I. (2016). 2016 update of the PRIDE database and its related tools. *Nucleic Acids Res.* 44 D447–D456. 10.1093/nar/gkv1145 26527722 PMC4702828

[B30] Walter J. Nagy L. Hein R. Rascher U. Beierkuhnlein C. Willner E. (2011). Do plants remember drought? Hints towards a drought-memory in grasses. *Environ. Exp. Bot.* 71 34–40. 10.1016/j.envexpbot.2010.10.020

[B31] Wang W. Vignani R. Scali M. Cresti M. (2006). A universal and rapid protocol for protein extraction from recalcitrant plant tissues for proteomic analysis. *Electrophoresis* 27 2782–2786. 10.1002/elps.200500722 16732618

[B32] Wedeking R. Mahlein A.-K. Steiner U. Oerke E.-C. Goldbach H. E. Wimmer M. A. (2017). Osmotic adjustment of young sugar beets (*Beta vulgaris*) under progressive drought stress and subsequent rewatering assessed by metabolite analysis and infrared thermography. *Funct. Plant Biol.* 44 119–133. 10.1071/FP16112 32480551

[B33] Wedeking R. Maucourt M. Deborde C. Moing A. Gibon Y. Goldbach H. E. (2018). 1H-NMR metabolomic profiling reveals a distinct metabolic recovery response in shoots and roots of temporarily drought-stressed sugar beets. *PLoS One* 13:e0196102. 10.1371/journal.pone.0196102 29738573 PMC5940195

