# Supplementary figures and images for: A Protein-Linger Strategy Keeps the Plant On-Hold After Rehydration of Drought-Stressed Beta vulgaris
Source: Front Plant Sci. 2019 Mar 29;10:381. doi: 10.3389/fpls.2019.00381 (PMC6449722; doi:10.3389/fpls.2019.00381)

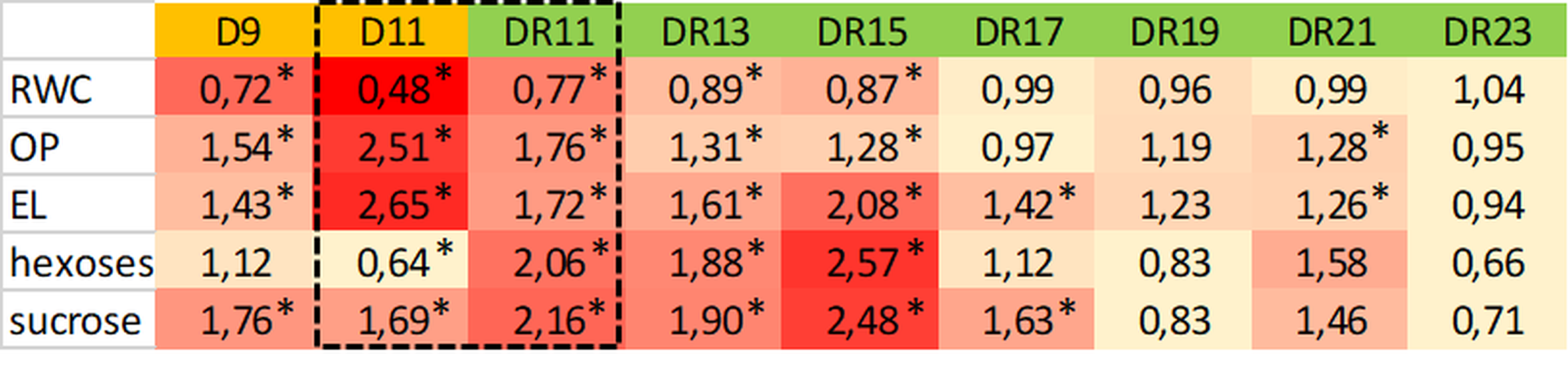

Supplement: FIGURE S1 — Time course of different physiological parameters (RWC: relative water content; OP: osmotic potential; EL: electrolyte leakage) and metabolites (hexoses: sum of fructose and glucose) during stress and recovery. Values represent ratios of stressed to control plants of the same day. Asterisks indicate statistically significant differences (controls versus treatment), ∗p < 0.05 (Kruskal-Wallis), n = 4. [file Image_1.TIF]

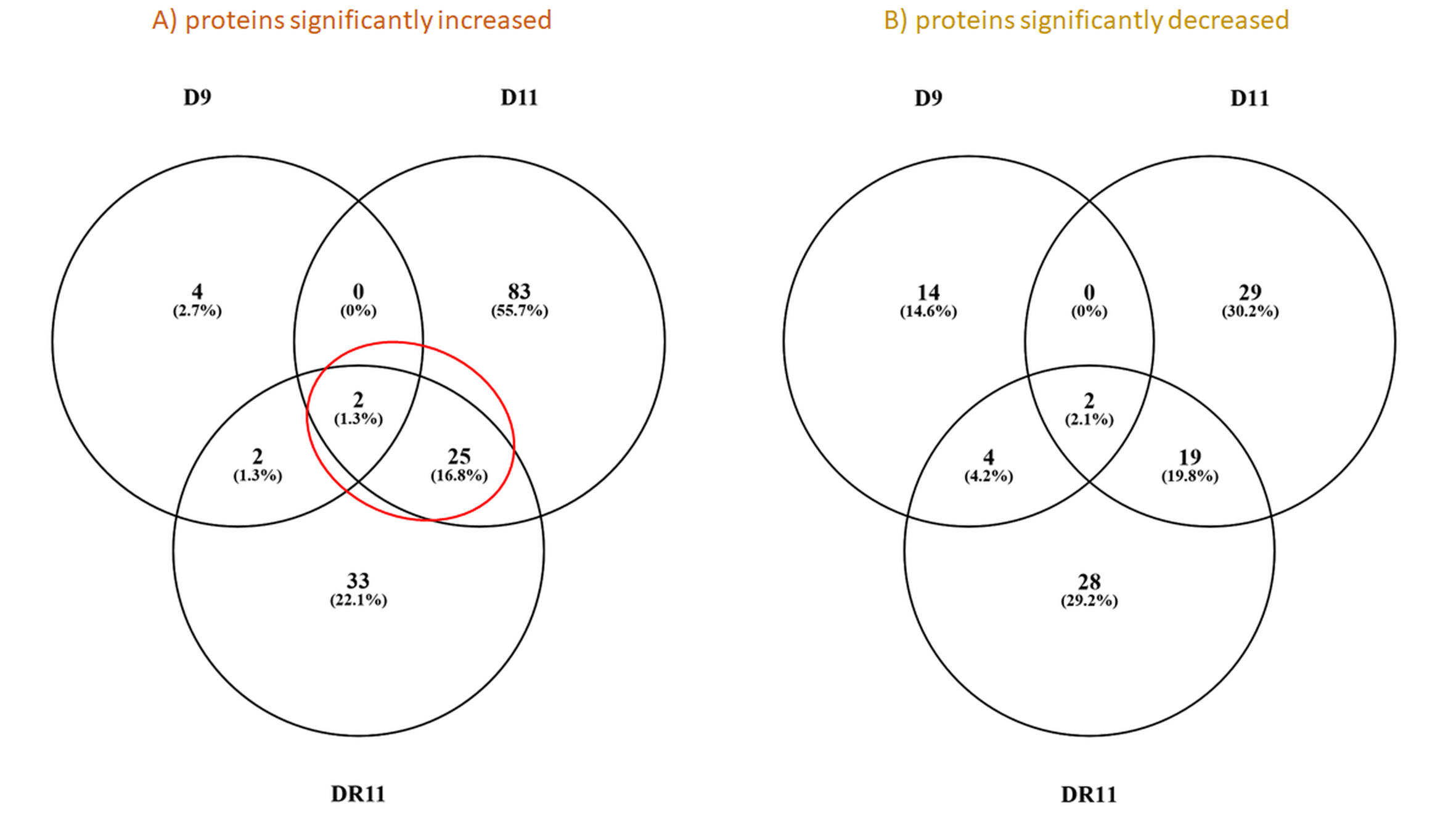

Supplement: FIGURE S2 — Venn diagrams of the numbers of statistically (p < 0.05; Kruskal-Wallis) significantly changed proteins overlapping between D9, D11 and DR11. [file Image_2.TIF]
